# Smokeless tobacco consumption and its association with risk factors of chronic kidney disease in rural and peri-urban Bangladesh

**DOI:** 10.18332/tid/171358

**Published:** 2023-10-20

**Authors:** Mohammad H. R. Sarker, Michiko Moriyama, Hasnat Sujon, Md M. Rahman, Shakila Banu, Mohammod J. Chisti, Tahmeed Ahmed, Abu S. G. Faruque

**Affiliations:** 1 Technical Training Unit, International Centre for Diarrheal Disease Research, Bangladesh, Dhaka, Bangladesh; 2 Graduate School of Biomedical and Health Sciences, Hiroshima University, Hiroshima, Japan; 3 Directorate General of Health Services, Ministry of Health and Family Welfare, Dhaka, Bangladesh; 4 Nutrition and Clinical Services Division, International Centre for Diarrheal Disease Research, Bangladesh, Dhaka, Bangladesh

**Keywords:** Bangladesh, chronic kidney disease, smokeless tobacco, tobacco

## Abstract

**INTRODUCTION:**

Compared to smoking, which has major consequences in chronic kidney disease (CKD) initiation and progression, smokeless tobacco (SLT) consumption is considered to have fewer health consequences. We investigated the prevalence of SLT consumption and its association with risk factors of CKD in a rural and peri-urban Bangladeshi population.

**METHODS:**

Using random sampling we recruited 872 adults in 2020, from the Mirzapur Demographic Surveillance System of Bangladesh, who had resided in the area for at least five years. Interviews using a semi-structured questionnaire, physical examination and anthropometric measurements were done, followed by blood and urine testing. The blood and urine tests were repeated in selected participants after three months as per the CKD Epidemiology Collaboration equation.

**RESULTS:**

The prevalence of SLT consumption was 29%. Being aged ≥46 years (OR=7.10; 95% CI: 4.79-10.94), female (OR=1.64; 95% CI: 1.21–2.22), housewife (OR=1.82; 95% CI: 1.35–2.45), farmer (OR=1.71; 95% CI: 1.06–2.76), widow (OR=3.40; 95% CI: 2.24–5.17), and having no formal schooling (OR=4.91; 95% CI: 3.59–6.72), family income of <$100/month (OR=1.66; 95% CI: 1.13–2.43), sleeping duration <7 hours per day (OR=2.33; 95% CI: 1.70–3.19), were associated with a significantly higher odds of SLT consumption. However, being aged 31–45 years (OR=0.25; 95% CI: 0.16–0.38) had significantly lower odds of being an SLT consumer. Among the diseases investigated, undernutrition (OR=1.63; 95% CI: 1.15–2.33), hypertension (OR=1.52; 95% CI: 1.13–2.05), anemia (OR=1.94; 95% CI: 1.39–2.71) and CKD (OR=1.62; 95% CI: 1.15–2.27) were significantly associated with SLT consumption. In the multivariable analysis, being aged 31–45 years (AOR=3.06; 95% CI: 1.91–4.90), ≥46 years (AOR=15.69; 95% CI: 4.64–53.09) and having no formal schooling (AOR=2.47; 95% CI: 1.72–3.55) were found to have a significant association with being an SLT consumer.

**CONCLUSIONS:**

SLT consumption is associated with most of the established risk factors of CKD within the studied population.

## INTRODUCTION

Chronic kidney disease (CKD) is a pressing public health concern in developed and developing nations, with approximately 700 million new cases diagnosed annually^1^. By 2030, an estimated 5–10 million CKD cases will necessitate costly interventions such as renal replacement therapy globally^2^. In developing countries such as Bangladesh, Pakistan, Brazil, and Indonesia, a variable prevalence of CKD has been reported, with over 10 million cases each year^1^. The absence of early diagnosis in developing countries limits timely treatment administration, placing patients at increased risk for expensive interventions. Therefore, identifying associated factors and implementing effective interventions to thwart those factors can be a primary means of preventive strategies to reduce the burden of morbidity and mortality. Age, diabetes, hypertension, obesity, high-sodium diets, and lead exposure are among the key risk factors for CKD^1^. Smoking is also significantly associated with CKD^3^. Although it is commonly believed that smokeless tobacco (SLT) consumption is less detrimental to health than smoking^4^, a study conducted by the authors of this article has identified SLT consumption as a risk factor for CKD with higher odds^5^.

Consumption of SLT has been prevalent worldwide for centuries, with dried tobacco leaves or powdered forms being chewed or sniffed. However, South-East Asia accounts for over 80% of global SLT consumption, with Bangladesh being the second largest consumer of SLT in the world, after India^6^. Unlike smoking, SLT consumption is socially acceptable among all classes in Bangladesh, where anti-tobacco programs focus mainly on smoking. This, combined with easy availability, low cost, and lack of awareness, has contributed to the persistence of high SLT consumption rates in the country^7^. Despite a slow decline in SLT consumption from 2009 to 2017 in Bangladesh, the number of deaths attributed to SLT consumption has increased^8^. SLT contains over 4000 chemicals, of which more than 30 are carcinogens, such as nitrosamines. SLT consumption has been reported to be associated with different forms of cancer, cardiovascular disease, and adverse pregnancy outcomes^9^. Furthermore, the use of SLT has been linked to kidney pathology in experimental studies^10^.

Many studies have been conducted in Bangladesh on SLT^11–13^. These studies mainly investigated the demographic characteristics of SLT consumers, the prevailing policy of the country related to SLT consumption, the reasons behind its consumption, the level of awareness among the general population and its association with different morbidities, particularly cancer and cardiovascular diseases. There is very limited evidence, if any, on the relationship between SLT consumption and CKD. This study aims to determine the prevalence of SLT consumption and its association with the established risk factors of CKD in rural and peri-urban areas of Bangladesh.

## METHODS

### Study site, participants, and sample size

**Figure 1:**
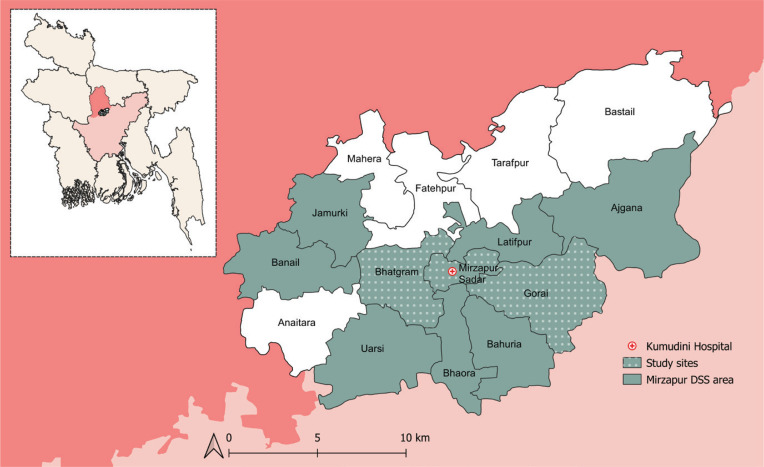
Map of Mirzapur subdistrict of Tangail district in Bangladesh. DSS, Demographic Surveillance Site. This cross-sectional study was conducted in the study sites of Mirzapur DSS in 2020 to assess the association of smokeless tobacco consumption with the risk factors of chronic kidney disease (N=872)

The methodology of this cross-sectional epidemiological study is described in detail elsewhere^5^. From January to June 2020, we conducted this study in the Mirzapur subdistrict of the Tangail district in Bangladesh. The area has a mixed rural and peri-urban population and comprises 15 unions (the smallest administrative division). The Mirzapur Demographic Surveillance System (DSS) is collecting routine demographic data from approximately 300000 people living in 10 unions of Mirzapur since its inception in 2007. Due to resource limitations, we purposively selected three unions (Mirzapur, Bhatgram, and Gorai) of Mirzapur DSS (Figure 1). A total of 872 adults (aged ≥18 years) who have been residents for at least five years were selected randomly from the DSS. However, participants hospitalized during the study period, and those with serious illnesses and provisionally diagnosed with a malignancy, mental illness, congenital disease, and/or physical disability were not included in our study.

### Data collection

The eligible participants in this study were visited at their homes by trained community healthcare workers who obtained written informed consent from the participants, administered a pre-tested structured questionnaire, performed physical examinations, and collected anthropometric measurements. Participants were advised to visit the diagnostic laboratory of Kumudini Hospital, and their blood and urine samples were collected to investigate morbidities such as diabetes, anemia, CKD, and lipid profile. Those who reported having estimated glomerular filtration rate (eGFR) <60 mL/min/1.73 m2 and/or albumin to creatine ratio ≥30 mg/g, were assessed again after three months as per the CKD Epidemiology Collaboration equation^14^. Their blood and urine samples were collected in the same hospital during the second assessment to evaluate their CKD status. Operational definitions of CKD, hypertension, diabetes, obesity, underweight, overweight, abdominal obesity, hypercholesterolemia, hypertriglyceridemia, anemia, undernutrition, family income, level of schooling, current tobacco smoker, and current SLT consumer, are described elsewhere^5^.

### Data analysis

We used SPSS v. 20.0 (IBM Co., Armonk, NY, USA) for statistical analysis and presented categorical variables as frequencies and percentages. Associations between outcome and exposure variables were examined using a two-tailed Pearson chi-squared test in the case of categorical variables. We performed multivariable logistic regression for variables with a p<0.05, and estimated the adjusted odds ratio (AOR) and 95% CI.

## RESULTS

Nearly half of the respondents were female, housewives, and aged ≥46 years. The majority were married, had some form of formal schooling and had a monthly family income of <$100. A considerable proportion of the participants reported consuming SLT (29%) and smoking (20%), with 5% reporting dual product use. Age-specific SLT consumption was 2%, 14%, and 43% for the age groups of 18–30, 31–45, and ≥46 years, respectively. Moreover, sex-specific SLT consumption was observed to be 33% and 23% for females and males, respectively. Additional risk factors obtained from physical examinations and laboratory investigations are presented in Table 1.

**Table 1:** Association of smokeless tobacco consumption with risk factors of chronic kidney disease in the cross-sectional study conducted in 2020 in Mirzapur, Tangail, Bangladesh (N=872)

*Variables*	*Smokeless tobacco consumer (N=252) n (%)*	*Smokeless tobacco non-consumer (N=620) n (%)*	*All (N=872) n (%)*	*OR (95% CI)*	*p*	*AOR^a^ (95% CI)*	*p*
**Age (years)^b^**							
18–30 (Ref.)	3 (1)	140 (23)	143 (16)	1		1	
31–45	31 (12)	186 (30)	217 (25)	0.25 (0.16–0.38)	<0.001	3.06 (1.91–4.90)	<0.001
≥46	218 (87)	294 (47)	512 (59)	7.10 (4.79–10.94)	<0.001	15.69 (4.64–53.09)	<0.001
**Gender^c^**							
Female	163 (65)	327 (53)	490 (56)	1.64 (1.21–2.22)	0.001	1.27 (0.59–2.74)	0.545
**Education level**							
No formal schooling	158 (63)	158 (25)	316 (36)	4.91 (3.59–6.72)	<0.001	2.47 (1.72–3.55)	<0.001
**Occupation**							
Housewife	150 (60)	277 (45)	427 (49)	1.82 (1.35–2.45)	<0.001	1.05 (0.42–2.65)	0.914
Farmer	31 (12)	47 (8)	78 (9)	1.71 (1.06–2.76)	0.037	1.27 (0.70–2.28)	0.427
**Marital status**							
Married	191 (76)	498 (80)	689 (79)	0.76 (0.54–1.09),	0.162	1.47 (0.90–2.39)	0.121
Widow	56 (22)	48 (8)	104 (12)	3.40 (2.24–5.17)	<0.001	3.02 (0.96–9.48)	0.058
**Family income**							
<$100/month	52 (21)	84 (13)	136 (16)	1.66 (1.13–2.43)	0.012	0.82 (0.52–1.28)	0.386
**Sleeping duration**							
<7 hours/day	102 (40)	140 (23)	242 (28)	2.33 (1.70–3.19)	<0.001	1.22 (0.85–1.74)	0.280
Present tobacco smoker	46 (18)	125 (20)	171 (20)	0.88 (0.61–1.29)	0.583	-	
**BMI (kg/m^2^)**							
Underweight (≤18.5)	34 (13)	57 (9)	91 (10)	1.54 (0.98–2.42)	0.078	-	
Overweight (25–29.9)	62 (25)	188 (30)	250 (29)	0.75 (0.54–1.05)	0.107	-	
Obese (≥30)	12 (5)	41 (7)	53 (6)	0.71 (0.36–1.38)	0.378	-	
Abdominal obesity^d^	94 (37)	247 (40)	341 (39)	0.89 (0.66–1.21)	0.535	-	
Undernutrition^e^	63 (25)	105 (17)	168 (19)	1.63 (1.15–2.33)	0.008	1.29 (0.84–1.97)	0.248
**Measurements**							
Hypertension^f^	121 (48)	234 (38)	355 (41)	1.52 (1.13–2.05)	0.006	0.95 (0.67–1.36)	0.790
Diabetes^g^	49 (19)	98 (16)	147 (17)	1.28 (0.87–1.87)	0.229	-	
Anemia^h^	79 (31)	118 (19)	197 (23)	1.94 (1.39–2.71)	<0.001	1.41 (0.96–2.07)	0.082
Chronic kidney disease^i^	71 (28)	121 (19)	192 (22)	1.62 (1.15–2.27)	0.006	0.92 (0.61–1.38)	0.685
Hypercholesterolemia^j^	59 (23)	128 (21)	187 (21)	1.17 (0.83–1.67)	0.417	-	
Hypertriglyceridemia^k^	93 (37)	193 (31)	286 (33)	1.29 (0.95–1.76)	0.117	-	

a AOR: adjusted odds ratio; adjusted for age, gender, education level, occupation, marital status, monthly family income, sleeping duration, undernutrition, hypertension, anemia, and chronic kidney disease.

b Age-specific smokeless tobacco consumers were 2%, 14% and 43% for the age group of 18–30, 31–45, and ≥46 years, respectively (n=872).

c Sex-specific smokeless tobacco consumers were 33% and 23% for females and males, respectively (n=872).

d Waist circumference ≥94 cm in males, and ≥80 cm in females.

e Mid-upper arm circumference <25 cm in males, and <24 cm in females.

f Currently require antihypertensive therapy, and/or systolic blood pressure ≥140 mmHg, and/or diastolic blood pressure ≥90 mmHg.

g History of diabetes, or fasting blood glucose >7 mmol/L.

h Blood haemoglobin <13 g/dL in males, and <12 g/dL in females.

i Measured according to the Chronic Kidney Disease Epidemiology Collaboration equation.

j Serum total cholesterol >200 mg/dL.

k Serum triglyceride >150 mg/dL.

In the univariate analysis, several factors were found to be significantly associated with SLT consumption: being aged ≥46 years (OR=7.10; 95% CI: 4.79–10.94), female (OR=1.64; 95% CI: 1.21–2.22), housewife (OR=1.82; 95% CI: 1.35–2.45), farmer (OR=1.71; 95% CI: 1.06–2.76), widow (OR=3.40; 95% CI: 2.24–5.17), having no formal schooling (OR=4.91; 95% CI: 3.59–6.72), family income <$100 per month (OR=1.66; 95% CI: 1.13–2.43), sleeping <7 hours per day (OR=2.33; 95% CI: 1.70–3.19), and experiencing health issues such as undernutrition (OR=1.63; 95% CI: 1.15–2.33), hypertension (OR=1.52; 95% CI: 1.13–2.05), anemia (OR=1.94; 95% CI: 1.39–2.71), and CKD (OR=1.62; 95% CI: 1.15–2.27). However, being aged 31–45 years was found to be protective against SLT consumption. Meanwhile, factors associated with CKD, such as being a current tobacco smoker, underweight, overweight, obese, having abdominal obesity, diabetes, hypercholesterolemia, and hypertriglyceridemia, did not significantly affect SLT consumption (Table 1).

In the multivariable analysis, while measuring the adjusted OR, the calculation was adjusted for age, gender, education level, occupation, marital status, monthly family income, sleeping duration, undernutrition, hypertension, anemia, and chronic kidney disease, all of which had significant unadjusted OR. Among the adjusted variables, being aged 31–45 years (AOR=3.06; 95% CI: 1.91–4.90), ≥46 years (AOR=15.69; 95% CI: 4.64–53.09), and having no formal schooling (AOR=2.47; 95% CI: 1.72–3.55) were found to be significantly associated with SLT consumption (Table 1).

## DISCUSSION

Overall, SLT consumption was found to be 29%, with the prevalence higher in females than in males. Being older, female, a farmer, housewife, widow, having no formal schooling, having low socio-economic status, less sleeping duration, undernutrition, hypertension, anemia, and CKD, had a significant association with being an SLT consumer.

Our measured prevalence of SLT consumption is higher than the national prevalence (23%) in rural Bangladesh^11^. The sex-specific distribution of SLT consumption also (33% among females and 23% among males) contrasts with the national average (25% among females and 16% among males)^11^. These differences could be attributed to the higher representation of females, illiterate individuals, and those aged ≥46 years in our study, all of which have been linked to higher SLT consumption. However, age-specific distribution of SLT consumption was comparable to the national distribution^11^. Our study revealed that SLT consumption is significantly linked to an age ≥31 years and being female. Age ≥60 years is considered a risk factor for CKD due to the natural decline in renal function in both sexes^1^. Regarding sex, while the risk of end-stage CKD is higher in males^15^, CKD is more prevalent in females^16^, with 1.29 times higher age-standardized prevalence in females^1^.

Education and SLT consumption share an inverse relationship^11^. We found that no formal schooling was significantly associated with SLT consumption. The relationship between lower levels of education and CKD may be due to various behavioral and sociodemographic factors, including poor dietary habits, lack of physical activity, and a higher prevalence of smoking^17^. In Bangladesh, individuals with limited education exhibit lower levels of health literacy, which can limit an individual’s ability to engage in self-care, make informed healthcare decisions, and adhere to medication regimes or lifestyle modifications to promote health^18^.

We have found higher odds of SLT consumption among farmers, housewives, widows, and people with low socio-economic status, although the association disappears in the multivariable model, possibly due to multicollinearity. Farmers and housewives, given their rural and peri-urban lifestyle, can consume SLT due to the common social acceptance of SLT consumption in the day-to-day culture of Bangladesh, in the same sense that a westerner drinks coffee^19^. It is usually thought that blue-collar workers are more prone to obesity, thereby to CKD, but as our study was in rural and peri-urban populations, we had a higher number of white-collar workers. A low-level occupation may accentuate the risk of CKD through exposure to hazardous working conditions, poor diet, and deleterious health behaviors. One study reported higher odds of SLT consumption among widows or divorced women^20^. As 12% of the widows in Bangladesh live a solitary life, they are prone to economic misfortune due to the absence of a steady income and have a stressful life, which may be the reason behind their consumption of SLT^21^. Following a 10-year observation period, a study in Iran found a significant association between CKD and individuals who were widowed or divorced^22^. On the other hand, low socio-economic status has been under scrutiny in several studies. The association between low-income status and CKD may be due to food and nutrition deficiency, anxiety, inadequate or inability to access (quality) healthcare, and other disease burdens. All of these can lead to unhealthy lifestyles, hypertension and diabetes, which have a causal link to CKD^23^.

Several studies have reported an association between poor sleep, low GFR, and the development of CKD. The cause of this is yet to be explained in detail, but it is hypothesized that sleep-wake rhythm and melatonin secretion has a relationship with kidney function regulation^24^. We observed that adults who slept <7 hours daily have higher odds of consuming SLT. A reciprocal relationship has been described with tobacco use and short sleep cycle^25^, which should be evaluated further.

Numerous animal and human studies have established a robust connection between smoking and CKD. Clinical evidence has demonstrated that smoking can exacerbate the progression of CKD. Animal models have demonstrated that nicotine can increase renal injury through various mechanisms, such as raising blood pressure, decreasing GFR, and increasing the effective renal plasma load ^26^. However, our study found no significant association between tobacco smoking and SLT consumption, despite the fact that 5% of the participants reported dual usage.

High body mass index (BMI) and abdominal obesity are associated with CKD^1^. However, no significant association was found between the consumption of SLT and BMI in our investigation. Similarly, dyslipidemia did not appear to be linked with SLT use. Conversely, our study found that undernutrition was significantly associated with SLT consumption, as reported in previous studies^27^. While most research on the relationship between tobacco use and appetite has focused on smoking, the association between tobacco consumption and undernutrition is well established. Nicotine, whether consumed through smoking or smokeless tobacco, activates brain nicotine receptors, which leads to decreased appetite^28^. Moreover, our study participants mainly came from rural areas and were economically disadvantaged. The financial burden caused by SLT consumption may hinder their ability to purchase healthy foods, leading to undernourishment. Recent research also indicates that Bangladeshi households using tobacco consume less milk, dairy products, and oil/fat than non-tobacco-using households^29^.

Among the major risk factors for CKD, our study revealed a significant association between SLT consumption and hypertension, while no significant relationship was found with diabetes. Previous studies have also reported a link between SLT consumption and hypertension^30^. This association can be attributed to nicotine and sodium, which are important constituents of SLT and have been implicated in hypertension^31^. Hypertension and CKD are closely intertwined pathophysiological states; sustained hypertension can lead to the development and progression of end-stage CKD, and conversely, a decline in renal function can lead to the development of hypertension. The association of SLT consumption with type 2 diabetes mellitus has been explored in several studies, and no significant association with a higher risk of diabetes has been reported^32^, although the pathophysiology of this disorder in relation to SLT consumption is yet to be described.

The relationship between anemia and CKD is intriguing. CKD reduces erythropoietin production, resulting in anemia. Anemia, in turn, exacerbates renal damage through hypoxia and indicates worse renal survival^33^. Our investigation also revealed a correlation between anemia and SLT consumption. More than 40% population of Bangladesh suffers from anemia^34^. Studies have reported that SLT consumption negatively correlates with almost all hematological parameters^35^.

A comprehensive analysis of the relationship between kidney ailments and SLT usage has not been undertaken. Nonetheless, our analysis revealed a greater odds ratio of CKD among SLT users. Several experimental studies have proposed a connection between kidney disorders and SLT consumption^36^. Further research in this area is warranted.

The Framework Convention for Tobacco Control (FCTC) has emerged as a pivotal global treaty to fight the tobacco epidemic, although the treaty focused mainly on smoking while neglecting SLT consumption. Bangladesh revised its tobacco control legislation in 2013 to include SLT consumption. Since risk mitigation is the most practical solution for CKD in developing nations, efforts to control SLT consumption should be part of public health strategies. As SLT consumption is deeply entrenched in cultural practices in countries like Bangladesh, Pakistan, and India, raising public awareness is also of paramount importance.

### Strengths and limitations

Our study has several strengths including unbiased random sampling with a large sample size, employment of accredited laboratories, and the use of validated and standardized equipment. Moreover, we employed the CKD Epidemiology Collaboration equation to measure GFR, with samples collected at three-month intervals. However, this equation was developed based on the population of the United States and may not be as suitable for the Bangladeshi population. The study’s main limitations were the convenient selection of three unions, the study being restricted to only one sub-district of the country, nutritional status assessment using only mid-upper arm circumference, and cross-sectional design. Reporting bias from the respondents’ end might also affect several variables. In addition, considering medications that might affect GFR/renal function was outside the scope of our study. Although data on the duration of SLT use, frequency and quantity of SLT consumption, and the quantification of the chemical compounds in the urine of the respondents could guide preventive approaches more accurately, these evaluations were also outside the scope of this study.

## CONCLUSIONS

Our study has provided initial evidence of an association between SLT consumption and several risk factors for CKD, including older age, illiteracy, shorter sleep duration, undernutrition, hypertension, and anemia. Although further research is warranted to elucidate the underlying mechanisms and confirm these findings, our study serves as a valuable resource for policymakers seeking to improve public health in rural and peri-urban regions of developing nations. Therefore, we recommend implementing interventions aimed at curbing SLT consumption on a larger scale.

## Data Availability

The detailed dataset is available from the correspondence author. A copy of the original data is also stored in the data archive of International Centre for Diarrheal Disease Research, Bangladesh. Non-identifiable data can be made accessible upon request, subject to the approval of the Research Administration department of International Centre for Diarrheal Disease Research, Bangladesh.
